# Polysorbate 80‐induced leaky gut impairs skeletal muscle metabolism in mice

**DOI:** 10.14814/phy2.14629

**Published:** 2020-10-28

**Authors:** Saho Nishimura, Wataru Aoi, Hinako Kodani, Yukiko Kobayashi, Sayori Wada, Masashi Kuwahata, Akane Higashi

**Affiliations:** ^1^ Division of Applied Life Sciences Graduate School of Life and Environmental Sciences Kyoto Prefectural University Kyoto Japan; ^2^ Division of Nutrition Management Osaka University Hospital Osaka Japan

**Keywords:** glucose metabolism, inflammation, intestinal permeability, mitochondria, skeletal muscle

## Abstract

Impaired intestinal permeability can induce systemic inflammation and metabolic disturbance. However, the effect of impaired intestinal permeability on metabolic function in the skeletal muscle is unknown. Dietary polysorbate 80 (PS80), a common emulsifier, has been shown to impair intestinal permeability in mice. Here, we investigated the effect of PS80‐induced intestinal permeability on glucose tolerance with metabolic signaling in the skeletal muscle. Male ICR mice were divided into control and PS80 groups. In the PS80 group, PS80 was contained in the drinking water at 1% (w/v). After 4 weeks, plasma fluorescein isothiocyanate (FITC) intensity was measured after orally administering FITC‐dextran. Half of the mice in each group underwent running exercises. Metabolic and inflammatory parameters were examined in the blood and skeletal muscle. Plasma FITC and lipopolysaccharide levels were higher in the PS80 group than the control group (*p* < .01, *p* = .085). The expression of tumor necrosis factor‐α in the skeletal muscle was increased upon PS80 administration (*p* < .05). Although the homeostasis model assessment ratio was higher in the PS80‐fed mice (*p* < .05), insulin‐signaling activity in the muscle did not differ between groups. Muscular pH, mitochondrial cytochrome oxidase activity, and glycogen content after exercise were lower in the PS80 group (*p* < .05) than the control group. There was a negative correlation between plasma FITC and muscle glycogen levels in the exercised groups (*r* = −.60, *p* < .05). These results suggest that daily PS80 intake induces intestinal permeability, leading to glucose intolerance and mitochondrial dysfunction in the skeletal muscle.

## Graphical abstract

This study investigated the effect of emulsifier‐induced leaky gut on metabolic response in skeletal muscle. The findings suggest that dietary polysorbate 80, a common emulsifier, caused intestinal permeability, leading to metabolic disturbance in sedentary and exercise conditions.

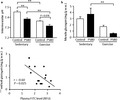

## 1 INTRODUCTION

The intestine plays important role in the digestion and absorption of nutrients. It also acts as a barrier by preventing the invasion of extrinsic factors such as bacteria, antigens, and endotoxins. The intestinal endothelium is composed of the “tight junction” structure between endothelial cells. Thus, in contrast to the transmembrane absorption of nutrients, these extrinsic factors cannot be transported via the paracellular space from the intestinal tract into the bloodstream. However, the destruction of the tight junction increases abnormal permeability and allows their invasion into the blood circulation. Accumulating evidence has shown that elevated intestinal permeability disturbs metabolic and immune functions, referred to as leaky gut syndrome (Peters & Wekerle, 2019; Sanz et al., 2015). Various dietary habits, including high‐fat diet, high‐sugar diet, and higher emulsifier consumption, can increase the risk of leaky gut syndrome (Chassaing et al., 2015; Moreira et al., 2012; Volynets et al., 2017).

Dietary polysorbate 80 (PS80) is an emulsifier commonly used in various processed foods. It has been shown that a continuous low dose of PS80 impairs intestinal permeability in mice (Chassaing et al., 2015). In addition, PS80 administration causes the development of obesity and metabolic dysfunction, associated with low‐grade inflammation and microbiota dysbiosis in the intestine (Chassaing et al., 2017). Thus, PS80‐induced intestinal permeability can expand to the impairment of whole‐body metabolism, potentially through leakage factors, such as bacteria and endotoxins. Additionally, it can be used as a diet‐induced leaky gut animal model.

The skeletal muscle functions as a major nutrient consumer—skeletal muscles consume >70% of blood glucose—and its proper metabolic functioning is a key factor that can prevent metabolic diseases (Egan & Zierath, 2013). It is well known that muscle metabolism dysfunction causes non‐communicable diseases, including type‐2 diabetes and dyslipidemia (Lavie et al., 2015; Milton et al., 2014). In addition, glucose and lipid metabolic functions also play a key role in maintaining muscle contraction (Hargreaves & Spriet, 2015). Therefore, muscle metabolic capacity influences endurance. These facts led us to hypothesize that intestinal permeability progression may decrease glucose tolerance and endurance associated with impaired glucose metabolism in the skeletal muscle. Here, we show systemic glucose intolerance with inactivating metabolic capacity of the skeletal muscle in the PS80‐induced leaky gut model.

## 2 MATERIALS AND METHODS

### 2.1 Animals and experimental design

The present study complied with the principles and guidelines of the Japanese Council on Animal Care and was approved by the Committee for Animal Research of the Kyoto Prefectural University (permission no. 290601). ICR mice (7 weeks old) were obtained from Shimizu Laboratory Supplies Co., Ltd. and acclimatized to an air‐conditioned (22 ± 2°C) room with a 12 hr light/dark cycle (lights on from 07:30 to 19:30 hr) for 1 week. The mice were divided into two groups: control group (*n* = 16) and PS80 administration group (*n* = 17). Mice in the PS80 group had free access to drinking water containing PS80 (1% w/v; Sigma), as mentioned in a previous study (Chassaing et al., 2015). The diet was identical for mice in both groups. After 4 weeks, an intestinal permeability test was conducted. In the next week, half of the mice from each group underwent running exercise for 30 min at 25 m/min. Following exercise, the mice were euthanized under anesthesia, and the interstitial pH in hind limb muscles was measured. Then, blood and gastrocnemius muscles were collected.

### 2.2 Intestinal permeability test

After starvation for 4 hr, the mice received a 3.2% fluorescein isothiocyanate (FITC)‐dextran (Sigma) by gavage at 50 µl/10 g body weight. After 1 hr, blood samples were collected from the tail vein.

### 2.3 pH measurements

The pH levels in the interstitial fluid of exercised muscle tissue were measured using a glass microelectrode under anesthesia (Aoi et al., 2015). The microprobe was inserted into the interstitium between the gastrocnemius and tibialis anterior muscles.

### 2.4 Blood analysis

Blood glucose levels were measured (GluTest; Arkray, Inc.), and then the blood samples were centrifuged at 3,500 *g* for 15 min at 4°C to collect plasma. Plasma insulin and lipopolysaccharide (LPS) were measured using test assay kits (Mercodia; Charles River Laboratories) according to the manufacturers' instructions.

### 2.5 Glycogen measurement

Muscle glycogen was isolated and purified by ethanol precipitation from a digest formed by the addition of potassium hydroxide solution, followed by quantification using the phenol‐sulfuric acid method (Aoi et al., 2011).

### 2.6 Protein analysis

Proteins were extracted from muscle tissues using a lysis buffer (Sigma) containing protease inhibitor (Sigma) and phosphatase inhibitor cocktails (Nacalai Tesque). Equal amounts of proteins in the lysates were separated by 10% sodium dodecyl sulfate‐polyacrylamide gel electrophoresis, which were then transferred onto nitrocellulose membranes. The blots were incubated with primary antibodies against phospho‐AMPKα (Thr172), total AMPKα, phospho‐Akt (Ser473), total Akt (Cell Signaling Technologies), and glyceraldehyde‐3‐phosphate dehydrogenase (GAPDH; Abcam). Subsequently, membranes were incubated with a horseradish peroxidase‐conjugated secondary antibody and visualized using an enhanced chemiluminescence substrate (Chemi‐Lumi One Super; Nacalai Tesque). Signal intensities were quantified using ImageJ software (National Institute of Health). Another sample set of tissue lysate was used to measure cytochrome oxidase activity with a test assay kit (Biovision) according to the manufacturer's instructions. The results were corrected with the total protein contents of each sample.

### 2.7 Real‐time PCR

Total RNA was extracted using Sepazol (Nacalai Tesque). After reverse transcription, quantitative PCR was performed using a Light Cycler 96 Real‐Time PCR system (Roche Life Science) with TaqMan PCR Master Mix and TaqMan primers (tumor necrosis factor α [TNFα]: ID Mm00443258_m1, GAPDH: ID Mm99999915_g1). Threshold cycle (C_t_) values were determined using Light Cycler Software, version 4 (Roche Life Science), and relative gene expression was calculated by the comparative C_t_ method using *GAPDH* as a reference gene.

### 2.8 Statistical analyses

All data are presented as the mean ± *SE*. Some measurements could not be completed for insufficient sample volume. Differences between groups were evaluated using a two‐way ANOVA or unpaired *t* test. If ANOVA indicated a significant difference, the Tukey–Kramer test was used to determine the significance of differences between means. *p* < .05 was considered to indicate statistical significance.

## 3 RESULTS

### 3.1 Body and tissue weights and intestinal permeability

**FIGURE 1:**
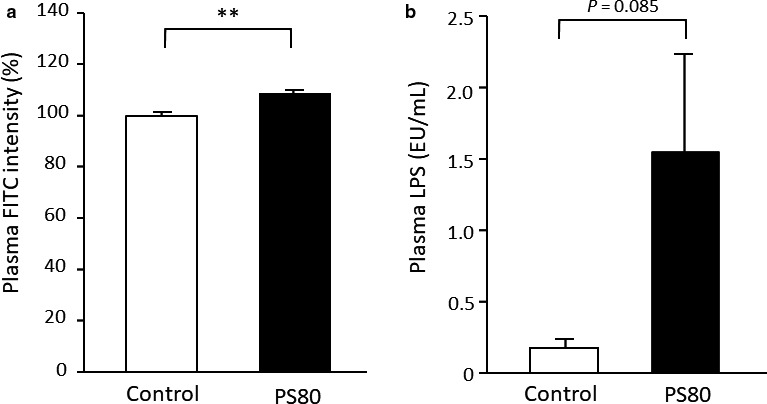
PS80 impaired intestinal permeability. (a) Plasma fluorescein‐isothiocyanate (FITC) intensity and (b) lipopolysaccharide (LPS). **Statistically significant differences were at the level of *p* < .01. Values are represented as the mean ± *SE*, with *n* = 7–8/group

Body, gastrocnemius muscle, and epididymal fat weights were unaltered between the control and PS80 groups. In the intestinal permeability test, plasma FITC intensity was higher in the PS80 group than in the control group (*p* < .01; Figure 1a). In addition, plasma LPS concentration was higher in the PS80‐administered mice than in the control mice, although this difference was close to statistical significance (*p* = .085; Figure 1b). These results suggest that chronic PS80 administration increased intestinal permeability.

### 3.2 Blood metabolic parameters

**FIGURE 2:**
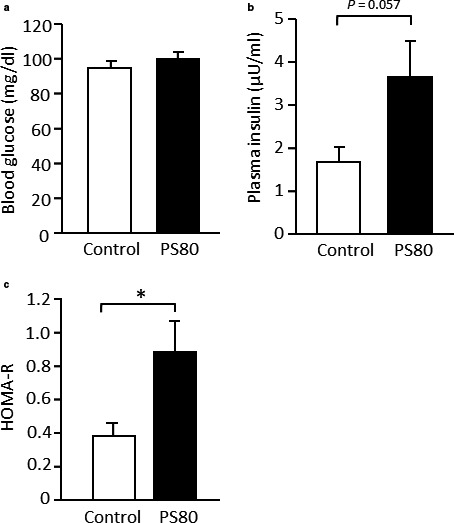
Blood metabolic parameters were impaired in PS80‐administered mice. (a) Blood glucose level, (b) plasma insulin level, and (c) homeostasis model assessment ratio (HOMA‐R). *Statistically significant differences were at the level of *p* < .05. Values are represented as the mean ± *SE*, with *n* = 8/group

There was no significant difference in blood glucose levels between the control and PS80 groups (Figure 2a). In contrast, plasma insulin levels were close to being significantly higher (*p* = .057; Figure 2b) in PS80‐administered mice. The homeostasis model assessment ratio (HOMA‐R) was significantly higher (*p* < .05; Figure 2c) in the PS80‐administered mice than in the control mice. This suggests that PS80‐administered mice had impaired insulin sensitivity.

### 3.3 Muscle metabolic and inflammatory parameters

**FIGURE 3:**
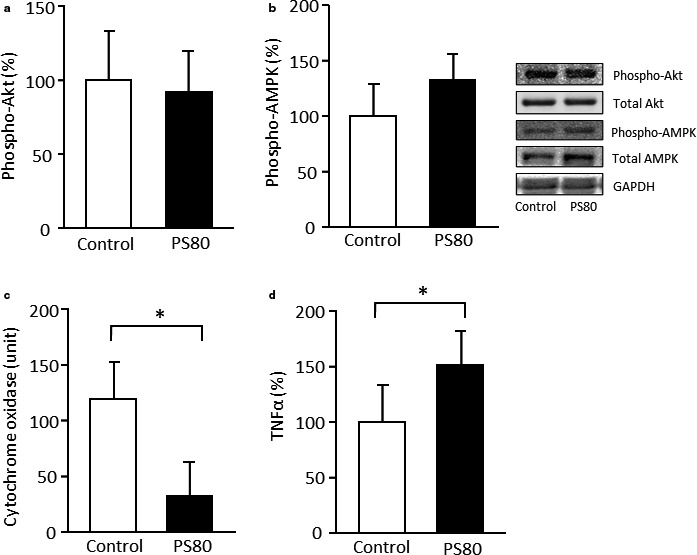
Metabolic and inflammatory responses in the skeletal muscle were impaired in PS80‐administered mice. Phosphorylation levels of (a) Akt and (b) AMP‐activated kinase (AMPK), (c) cytochrome oxidase activity, and (d) tumor necrosis factor‐α (TNF‐α) expression. *Statistically significant differences were at the level of *p* < .05. Values are represented as the mean ± *SE*, with *n* = 7–8/group

No significant differences in phospho‐Akt and phospho‐AMPK levels were found between the two groups (Figure 3a,b). In contrast, cytochrome oxidase activity decreased upon PS80 administration (*p* < .05; Figure 3c). In addition, the mRNA level of TNFα was higher in the PS80 group than in the control group (*p* < .05; Figure 3d).

**FIGURE 4:**
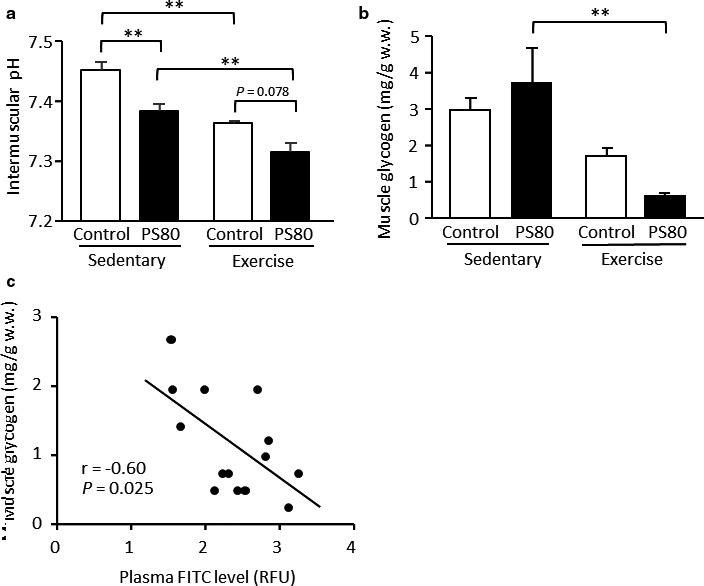
Exercise‐induced metabolic responses were impaired in PS80‐administered mice. (a) Interstitial fluid pH and (b) glycogen content in the gastrocnemius muscle. (c) A negative correlation between plasma fluorescein‐isothiocyanate (FITC) level and muscle glycogen content. **Statistically significant differences were at the level of *p* < .01. Values are represented as the mean ± *SE*, with *n* = 7–9/group

The pH level of muscle tissues post‐exercise is lactic acid‐generation dependent. Most lactate anions are either released into circulation or immediately metabolized as an energy substrate in the mitochondria. Owing to the limited buffering factors such as proteins, the buffering capacity is lower in the interstitial fluid than cytosol and blood. Therefore, the pH of the interstitial fluid in muscle tissues can rapidly change in response to muscle contraction and can be used as a stable marker of acid–base conditions and glycolysis activity. The interstitial pH levels in the muscle were reduced following exercise in both the control and PS80 groups (*p* < .01; Figure 4a). Lower pH levels were found in the PS80‐administered mice in both resting and exercised conditions. In addition, muscle glycogen content showed a significant decrease with exercise only in PS80‐administered mice (*p* < .01), although it did not differ between groups in the resting condition (Figure 4b). There was a significant negative correlation between plasma FITC and muscle glycogen levels in the exercised groups (*r* = −0.60, *p* < .05; Figure 4c).

## 4 DISCUSSION

Our study revealed that the daily administration of a common emulsifier, PS80, (a) impaired intestinal permeability; (b) elevated plasma endotoxin levels with higher TNFα expression in muscle tissues; and (c) disturbed muscular metabolic characteristics in resting and exercised conditions. Although increased intestinal permeability is known to disturb the metabolic function of the liver and adipose tissues (Kim et al., 2012; Ray & NAFLD., 2015), the effect of leaky gut syndrome on the skeletal muscle, a major nutrient consumer, remains unclear. The observations of this study provide the first evidence of a close association between intestinal permeability and metabolic functions in the skeletal muscle, at least in part, via the inflammatory response of invasion factors in circulation, thereby regulating metabolic health and athletic performance.

It has been suggested that increased consumption of emulsifiers in daily life can cause bacterial translocation across mucosal surfaces and elevate the incidence of inflammatory bowel diseases (Partridge et al., 2019). In animal models, chronic PS80 administration gradually elevates intestinal permeability, which progresses over 12 weeks (Chassaing et al., 2015). In the FITC‐dextran injection experiment, we found an increase in intestinal permeability after 4 weeks of PS80 administration. The dextran particles with no specific carriers for transport are scarcely transported via the intestinal tract into the bloodstream under normal conditions. Thus, the intestinal barrier prevents the translocation of bacteria and endotoxins into the body. However, these abnormal factors can be moved through the paracellular route when the tight junction is destroyed. In addition to higher FITC levels, we also found a nearly significantly higher circulating LPS level in PS80 administered condition, which supports the leaky gut condition. Some bacteria generate LPS in the intestinal tract. In addition, circulation‐translocated bacteria can also become a source of LPS generation. Such intestine‐derived contaminants can act as systemic pro‐inflammatory factors in various tissues (Leclercq et al., 2012; Little et al., 2018; Schietroma et al., 2013). In fact, we found elevated expression of TNFα, an inflammatory cytokine, in the skeletal muscle, suggesting low‐grade inflammation in the tissues.

Several studies have suggested that long‐term administration of PS80 shows a direct modification of microbiota in terms of composition and reduced diversity (Chassaing et al., 2015, 2017). As typical examples, reduced levels of favorable *Bacteroidales* and increased levels of mucolytic *Ruminococcus* were observed. The increase in the abundance of bacteria that can produce pro‐inflammatory factors, such as LPS, is associated with systematic inflammation (Salguero et al., 2019). Indeed, transplantation of feces obtained from PS80‐administered donor mice showed low‐grade inflammation in germ‐free mice (Chassaing et al., 2015). Consequently, PS80 decreases the mucus thickness and causes the destruction of tight junction proteins, thereby elevating intestinal permeability (Lock et al., 2018). Many experimental and epidemiological studies have supported the concept that microbiota dysbiosis is an underlying cause of inflammatory bowel diseases, which further causes leaky gut syndrome (Li et al., 2015; Michielan & D'Incà, 2015; Willing et al., 2010).

Growing evidence suggests that the leaky gut causes metabolic dysfunction. Elevated intestinal permeability accelerates fat accumulation and leads to obesity and hepatic steatosis (Kim et al., 2012; Ray & NAFLD., 2015). In metabolic abnormalities, dysregulation of metabolic signaling by chronic low‐grade inflammation is involved (McNelis & Olefsky, 2014). Transplantation of feces obtained from PS80‐administered donor mice showed metabolic dysfunction in germ‐free recipient mice (Chassaing et al., 2015). However, metabolic responses in the skeletal muscle of the leaky gut condition have been unclear. In this study, plasma insulin levels were non‐significantly higher in the PS80 group, while blood glucose levels were similar to those of the control group, resulting in a higher HOMA‐R level. In this case, insulin‐dependent and insulin‐independent signaling factors of glucose uptake in the muscle were not activated, suggesting muscle glucose intolerance in the leaky gut mice. However, insulin secretion was stimulated to maintain homeostasis of blood glucose levels and glucose uptake. In addition, we found mitochondrial metabolic dysfunction in PS80‐administered mice. The inhibitory effect of aerobic metabolism in the mitochondria causes energy supply through glycolysis, leading to glycogen utilization and lactic acid production. Indeed, the glycogen content and pH levels were lower in the PS80 group than in the control group following exercise, suggesting higher glycolytic activity in the skeletal muscle of leaky gut mice. Furthermore, the negative correlation between plasma FITC level and glycogen content post‐exercise supports the concept that leaky gut impairs aerobic metabolism.

Circulating factors may be involved in muscle metabolic dysfunction. We found higher levels of LPS in PS80‐administered mice. LPS is a typical factor that stimulates inflammatory responses through Toll‐like receptor 4 in various cell types. It is well known that LPS elevates the expression of inflammatory cytokines, such as TNFα and interleukin‐1β, in skeletal muscle cells (Frost et al., 2001). Inflammatory cytokines have been shown to impair insulin sensitivity by inactivating signaling factors such as insulin receptor substrate, PI3‐kinase, and Akt (Frost et al., 2001). Treatment with inflammatory cytokines decreases the insulin‐stimulated phosphorylation of these factors and subsequently glucose uptake (Alvaro et al., 2004). In addition, inflammatory cytokines also impair metabolic signal transduction and mitochondrial biogenesis (Sente et al., 2016). In this study, we observed elevated TNFα expression in the muscle of leaky gut mice. Unchanged phosphorylation levels of Akt even at higher plasma insulin levels suggest impaired insulin sensitivity. Furthermore, the activity of cytochrome oxidase, a key metabolic enzyme in mitochondria, was lower in the muscle. A factor that may cause muscle inflammation is inflammatory cytokines derived from adipose tissue. Indeed, a positive relationship between circulating inflammatory cytokine levels and body fat accumulation is well known (Hosseinzadeh‐Attar et al., 2016; Kahn et al., 2006). However, no significant increase in adipose tissue was found at this time point. Therefore, LPS‐induced inflammation might lead to glucose intolerance and mitochondrial aerobic metabolism in the PS80 group. Other circulating factors, such as zonulin, fatty acid‐binding protein‐2, and flagellin, elevated in the leaky gut condition, are also involved in metabolic impairment (Fukui, 2016). Further investigation is necessary to determine the mechanisms underlying the relationship between intestinal permeability and metabolic dysfunction.

In the present study, we used the PS80‐induced leaky gut model to investigate the relationship between intestinal permeability and muscle metabolism. However, it is unclear whether this is observed in other leaky gut conditions. For example, other emulsifiers, such as carboxymethylcellulose, also impair intestinal permeability, while the speed of permeability progression and changes in gut microbiota are different from the PS80‐administered condition (Chassaing et al., 2015, 2017). In addition, high‐fat diet, high‐sugar diet, and medications have been shown to impair intestinal permeability (Hamada et al., 2010; Moreira et al., 2012; Volynets et al., 2017). In the future, it is necessary to examine whether they impair muscle metabolism in a similar manner.

In conclusion, daily intake of PS80 for 4 weeks impaired intestinal permeability, thereby inducing leaky gut. Muscle TNFα levels were elevated with a nearly significant circulating LPS increase in the PS80 group. Although HOMA‐R was higher in the PS80‐administered mice, insulin‐dependent signaling in the skeletal muscle did not differ between the two groups. Muscular pH and glycogen levels after exercise were lower in the PS80 group, along with reduced mitochondrial cytochrome oxidase activity. These results suggest that PS80‐induced leaky gut causes glucose intolerance and mitochondrial dysfunction in the skeletal muscle, which is mediated by inflammatory responses and involves alterations in the “muscle‐gut axis.”

## CONFLICT OF INTEREST

None declared.

## AUTHOR CONTRIBUTION

S.N. and W.A. designed and coordinated the study. S.N., W.A., and H.K. contributed to experimental design and performance. S.N., W.A., H.K., Y.K., and M.K. analyzed and evaluated data. A.H. and S.W. supported the design and coordination. S.N. and W.A. wrote the manuscript with input from other authors. All authors critically reviewed and approved the final version of the manuscript.

## ETHICAL STATEMENT

All procedures were performed according to the guidelines and with the approval of the Committee for Animal Research of the Kyoto Prefectural University.

## Data Availability

Data reported in this manuscript will be made available upon reasonable request.
